# Supplementary material for: Semantic similarity across languages reflects neurocognitive dimensions shaped by climate
Source: Nat Commun. 2026 Mar 16;17:4016. doi: 10.1038/s41467-026-70608-8 (PMC13136401; doi:10.1038/s41467-026-70608-8)
Supplement: Supplementary file 1 — Supplementary Information [file 41467_2026_70608_MOESM1_ESM.pdf]

Supplementary Information for

Semantic similarity across languages reflects neurocognitive dimensions shaped by climate

Authors: Ze Fu, Yuxi Chu, Tangxiaoxue Zhang, Yawen Li, Xiaosha Wang*, Yanchao Bi*

***Corresponding authors:** Xiaosha Wang; Yanchao Bi
Email: xiaosha_wang@pku.edu.cn (XW); ybi@pku.edu.cn (YB)

The PDF file includes:

Supplementary Notes 1 to 6
Figures S1 to S9
Tables S1 to S7
Supplementary References

Supplementary Note 1

Principal component analyses results

In addition to inter-language correlation analyses, we conducted a Principal Component Analysis (PCA) on 204 concepts common across all 53 languages to further assess semantic commonalities. This complementary approach treats languages as features and semantic patterns as samples (Figure S2a), allowing us to extract the first principal component (PC1) as an indicator of cross-linguistic commonalities. The variance explained by PC1 reflects the degree of universality in semantic structure[1,2]. While both PCA and inter-language correlation methods aim to quantify cross-linguistic similarities, they differ in their approach to handling between-concept variations and the number of concepts analyzed. The PCA utilized a subset of 204 words shared across all 53 languages, concatenating dimensional vectors for each concept to construct comprehensive semantic structures.

Results (Figure S2b, upper panel) showed that the neurocognitive-dimensional representation exhibits the highest degree of universality (44.31%), followed by local-distributed (36.38%), feature-based (34.45%), and global-distributed (34.16%) models. Compared to statistical random models, only the neurocognitive model was consistently positioned at the upper bounds of the distributions, signifying a relatively universal semantic structure ($P < 0.001$ and $P = 0.009$, respectively; Figure S2b, lower panel).

For the neurocognitive semantic representation, the scree plot (Figure S2b, upper panel) showed a dominant first component, with subsequent components explaining substantially less variance (e.g., PC2 explains 4%). PC1 showed positive correlations with all 53 languages ($M = 0.66$, $SD = 0.13$), contrasting with PC2's mixed positive and negative loadings ($M = -0.02$, $SD = 0.20$). Spanish demonstrated the highest PC1 correlation ($r = 0.83$), while Korean exhibited the lowest ($r = 0.35$; full results in Figure S2c). The PC1 space of the neurocognitive structure matrix (Figure S2d) showed a general division between sensory-motor-related and social-cognitive-related dimensions. Notably, the *sound* dimension clustered with social-cognitive dimensions, while the *space* dimension aligned with sensory-motor dimensions. The matrix also visualizes cross-linguistic agreement in the representation of specific concepts, such as the strong association of *star* across shape, color, and time dimensions.

Supplementary Note 2

Validation using pretrained embedding models from Wikipedia and the Open Subtitles

To validate the findings from Study 1 (language computation study), we conducted additional analyses using text embedding data derived from a combination of Wikipedia and the Open Subtitles database[3]. This validation study examined semantic representations of 1,016 NorthEuralex (NEL) concepts across 35 languages, analyzing commonalities and variations in different semantic representations.

Language sample selection followed a two-step process: First, we identified 43 languages with translated word forms available in the word embedding models. Second, we excluded 8 languages that were missing over 25% vector representations for NEL concepts, ensuring adequate shared concept coverage between language pairs. This process resulted in a final sample of 35 languages.

We replicated the analysis of commonalities captured by neurocognitive semantic structures, constructing different theoretical models (neurocognitive, distributional local/global, and featural)

using semantic projection methods. Cross-language commonalities were compared using two complementary methods: inter-language correlation and principal component analysis.

As shown in Figure S4a (left panel), the neurocognitive-dimensional semantic representation demonstrated greater cross-language similarity compared to other representation types (mean Fisher z-transformed correlation: neurocognitive (M = 0.77, SD = 0.15); distributional (global) (M = 0.53, SD = 0.13); distributional (local) (M = 0.44, SD = 0.09); semantic feature (M = 0.43, SD = 0.11); Wilcoxon signed-rank tests: neurocognitive vs. distributional (global), V = 177309, P < 0.001; neurocognitive vs. distributed (local), V = 177310, P < 0.001; neurocognitive vs. semantic feature, V = 177310, P < 0.001).

We conducted a PCA for 35 languages and 354 NEL concepts shared across all languages. The variance explained by the first component (PC1) indicated that neurocognitive-based representations of concepts are more universal than other semantic structures (Figure S4a, right panel). The neurocognitive-dimensional representation exhibited the highest degree of universality (65.52%), followed by local-distributed (55.99%), global-distributed (49.66%), and feature-based models (47.55%). For the neurocognitive semantic representation, the scree plot showed a dominant first component, with subsequent components explaining substantially less variance (e.g., PC2 explains 3.4%). PC1 showed positive correlations with all 35 languages (M = 0.81, SD = 0.07), contrasting with PC2's mixed positive and negative loadings (M = -0.001, SD = 0.19).

Within a sample of 17 languages (136 language pairs) where all four environmental variables were available, they collectively explained 64% of the semantic (neurocognitive) space variations (Spearman ρ = 0.81, P < 0.001, 95% CI [0.74, 0.86]; Figure S4b). We replicated the association patterns between climate and neurocognitive semantic variations using a linear regression model to assess the unique effect of each environmental factor. As indicated in Figure S4b, climate showed the strongest unique explanatory effects (β = 0.44, P < 0.001, 95% CI [0.33, 0.58]). Culture and linguistic history also showed significant unique effects (culture: β = 0.22, P < 0.001, 95% CI [0.12, 0.33]; linguistic history: β = 0.28, P < 0.001, 95% CI [0.20, 0.35]). Geographic distance showed negative effects on semantic variations (β = -0.18, P = 0.002, 95% CI [-0.29, -0.06]). The effects of climate on specific neurocognitive dimensions were also replicated (12 of 13 semantic dimensions, FDR-corrected Ps < 0.05; shape, FDR-corrected P = 0.059; Figure S4b, right panel).

Supplementary Note 3

Statistical methods for controlling non-independence

In our study, we addressed potential biases arising from non-independent sampling of languages and subjects, particularly the spatial autocorrelations between samples, when estimating the effects of environmental variables. A primary concern was the non-independence in language sampling. While we incorporated other environmental variables into the same regression models as the potential controls, we implemented additional measures to ensure the robustness of our findings, particularly regarding the effects of environmental variables such as climate.

Linear mixed effects regression models. To account for the non-independent sampling of languages, we employed linear mixed regression models with a crossed random-effects structure. This approach nested language pairs within language families. Following the method suggested by Chen et al.[4], we doubled the data (with redundancy) to allow for fully crossed random effects, accounting for the symmetric nature of the inter-language/inter-subject matrix and the fact that each participant contributes twice in the model. Therefore, prior to statistical inference, we manually

adjusted the degrees of freedom to N - k, where N represents the number of unique observations and k denotes the number of fixed effects in the model. This correction ensures appropriate statistical power and reduces the risk of Type I errors. All reported findings in this article utilize these corrected degrees of freedom. The results of linear mixed effects regression models are presented in the main text.

Additional validation approaches. To further validate the robustness of the climate-semantics relationship, we implemented several complementary analytical strategies:

Alternative random effect structures: We tested more complex random effect specifications to rigorously control for hierarchical non-independence relationships. The enhanced model with nested random effects can be formally expressed as:

$$\text{Semantic\_Distance}_{ij} = \beta_0 + \beta_1 \text{Climate}_{ij} + (1 \mid \text{Family}_i/\text{Language}_i) + (1 \mid \text{Family}_j/\text{Language}_j) + \varepsilon_{ij} \quad (1)$$

, where i and j index the language pairs, β_0 represents the intercept, β_1 represent the fixed effects for climate, ($1 \mid \text{Family}_i/\text{Language}_i$) and ($1 \mid \text{Family}_j/\text{Language}_j$) represent the nested random intercepts where languages are nested within their respective language pairs (Language_{ij}) and language families (Family_{ij}), and εij represents the residual error term. This nested structure accounts for the hierarchical nature of the data, where language pairs belong to specific language families, thus more rigorously controlling for phylogenetic non-independence in the data.

These models consistently demonstrated significant climate effects across all three studies (Study 1 – Wikipedia + CC: β = 0.26, P < 0.001, 95% CI [0.20, 0.31]; Study 1 – Wikipedia + Subs: β = 0.44, P < 0.001, 95% CI [0.34, 0.54]; Study 2: β = 0.14, P = 0.004, 95% CI [0.05, 0.24]; Study 3: β = 0.12, P < 0.001, 95% CI [0.06, 0.19]), confirming that our findings are not artifacts of model specification.

Individual-level analysis. For Studies 2 and 3, we performed analyses at the individual subject dyad level rather than the language level. The model with the nested random effect's structure at this finer-grained level can be formally expressed as:

$$\begin{aligned}\text{Semantic\_Distance}_{ij} = \beta_0 + \beta_1 \text{Climate}_{ij} &+ (1 \mid \text{Family}_i/\text{Language}_i/\text{Subject}_i) \\ &+ (1 \mid \text{Family}_j/\text{Language}_j/\text{Subject}_j) + \varepsilon_{ij}\end{aligned} \quad (2)$$

, where i and j index the individual subject pairs, β_0 represents the intercept, β_1 represents the fixed effect for climate, ($1 \mid \text{Family}_i/\text{Language}_i/\text{Subject}_i$) and ($1 \mid \text{Family}_j/\text{Language}_j/\text{Subject}_j$) represent the three-level nested random effects structure where subjects are nested within languages, which are in turn nested within language families. This structure captures the complete hierarchical nature of our data by accounting for non-independence at the subject level (participants who speak the same language), the language level (languages that belong to the same family), and the language family level. These analyses showed significant climate effects (Study 2: β = 0.18, P < 0.001, 95% CI [0.17, 0.18]; Study 3: β = 0.07, P < 0.001, 95% CI [0.03, 0.11]), confirming that our findings persist at multiple levels of analysis.

Supplementary Note 4

Validation of commonalities in Study 2 and Study 3

To validate commonalties in neurocognitive dimensional structures shared among individuals in different language groups, we employed a variation comparative analysis framework on within-language and cross-language subject pairs using human rating data (Study 2) and neural activation data (Study 3).

Study 2: Validation of commonalities (human rating data)

Methods: We constructed an inter-subject correlation matrix using Pearson correlation coefficients between subjects' ratings on 207 concepts across 13 dimensions (2,691 ratings per subject). To examine the existence of a language-specific component, we conducted an independent Wilcoxon signed-rank test (two-sided) comparing rating similarities between within-language and cross-language subject pairs. We then applied the mean correlation method[2] to quantify commonalities and variations across languages. The variance of this matrix was decomposed into 3 distinct components: a universal component shared by individuals regardless of their spoken language, a language-specific component that represents the average of unique semantic knowledge shared by individuals within a particular language group, and a component that captures individual variance not accounted for by language-specific variations. The square root of the mean correlation of certain subject pairs captures the proportion of knowledge shared among a specific group. We thus performed the following calculations: (i) compute the square root of averaged Pearson correlation of cross-language subject pairs, as measurement of a shared proportion (denoted by $Var^2_{language\text{-}shared}$); (ii) compute the square root of averaged correlation of within-language subject pairs for each language (denoted by $Var^2_{shared\text{-}within}$), then subtract the shared proportion from sample-size-weighted mean of within-language knowledge, as measurement of a language-specific proportion ($Var^2_{shared\text{-}within}$ – $Var^2_{language\text{-}shared}$, denoted by $Var^2_{language\text{-}specific}$); and (iii) compute the individual variability portion by subtracting the shared proportion and language-specific variations from one (1 – $Var^2_{language\text{-}shared}$ – $Var^2_{language\text{-}specific}$, denoted by $Var^2_{individual}$).

Results: We investigated the existence of a language-specific component by comparing within-language similarities (N = 3,882 pairs) to cross-language similarities (N = 27,996 pairs). Results showed higher within-language similarities (M = 0.55) compared to cross-language similarities (M = 0.47; two-sided Wilcoxon rank-sum test, W = 71,836,161, P < 0.001). Our results further revealed a substantial shared proportion ($Var^2_{language\text{-}shared}$ = 65.78%), estimated from the square root of averaged correlation among cross-language pairs. The language-specific component ($Var^2_{language\text{-}specific}$ = 4.20%) was comparatively small, calculated as the difference between the square root of averaged within-language correlations and the shared component. The remaining variance ($Var^2_{individual}$ = 30.02%) was attributed to individual differences and sampling error.

Study 3: Validation of commonalities (neural activation data)

Methods: To assess the presence of language-specific components in neural representations, we conducted a comparative analysis of within-language and cross-language neural similarities similar to Study 2. Inter-subject correlation matrices were constructed from individual t-value maps for the previously identified ROIs. We employed a two-sided Wilcoxon signed-rank test to compare neural similarities between within-language subject pairs and cross-language subject pairs. Due to the shallow sampling approach used in the dataset (one or two speakers per language), precise estimation of the variance in commonalities and variations was not feasible.

Results: To assess the commonality of neural activity in our ROIs, we compared cross-language similarity (N = 3,614 pairs) with within-language similarity (N = 41 pairs). Results showed no significant differences between within-language and between-language pair similarities in the right ATL region:

M_{within} = 0.21, $M_{between}$ = 0.20, two-sided Wilcoxon rank-sum test W = 284356, P = 0.539, suggesting a relatively common nature of neural representations in these regions. This finding aligns with previous analyses of the whole language network[5].

Supplementary Note 5

Environmental prediction results of the right ATL in nonlinguistic tasks

To determine whether the observed climate effects on neural patterns were specific to language processing or reflected more general environmental influences on brain activity, we conducted control analyses using neural data from nonlinguistic cognitive tasks. We examined the same participants' neural responses in the right anterior temporal lobe (r-ATL) during two control tasks: Spatial Working Memory (Hard vs Easy contrast) and Math (Hard vs Easy contrast).

For each control task, we constructed neural representational dissimilarity matrices (RDMs) following the same procedures used for the language task and tested whether environmental variables (climate, cultural, geographic, and linguistic distances) predicted neural pattern dissimilarities using linear mixed-effects regression models with random intercepts for language families.

For the Spatial Working Memory task (Figure S7a), we found that cultural distance significantly predicted neural pattern dissimilarities (β = 0.13, P = 0.016, 95% CI [0.02, 0.24]), while climate distance showed no significant effect (β = 0.06, P = 0.213, 95% CI [-0.05, 0.16]). Geographic distance (β = -0.03, P = 0.501, 95% CI [-0.10, 0.05]) and linguistic distance (β = -0.003, P = 0.925, 95% CI [-0.07, 0.07]) also showed no significant effects. For the Math task (Figure S7b), none of the environmental variables significantly predicted neural pattern dissimilarities (all Ps > 0.05).

These results stand in contrast to our findings from the language task, where climate distance was the only significant predictor of neural pattern dissimilarities in the r-ATL. The dissociation between language and nonlinguistic tasks suggests that the climate effects observed during language processing are specific to linguistic mechanisms rather than reflecting general effects of environment on neural activity.

Supplementary Note 6

Multiple proofreading translation validation

First, professional translation from English to the target languages (Version A) was conducted. Version A was then back-translated into English using both ChatGPT (https://chat.openai.com) and deep-L Translate (https://www.deepl.com) to allow for direct comparison with the original version. A skilled bilingual individual proofread Version A and addressed any inconsistencies identified during the back translation, leading to the development of Version B. Version B was then submitted to a second professional translator for review, focusing on resolving previously identified corrections. This iterative process was repeated 1 to 3 times for each language until a consensus was reached on the final translation.

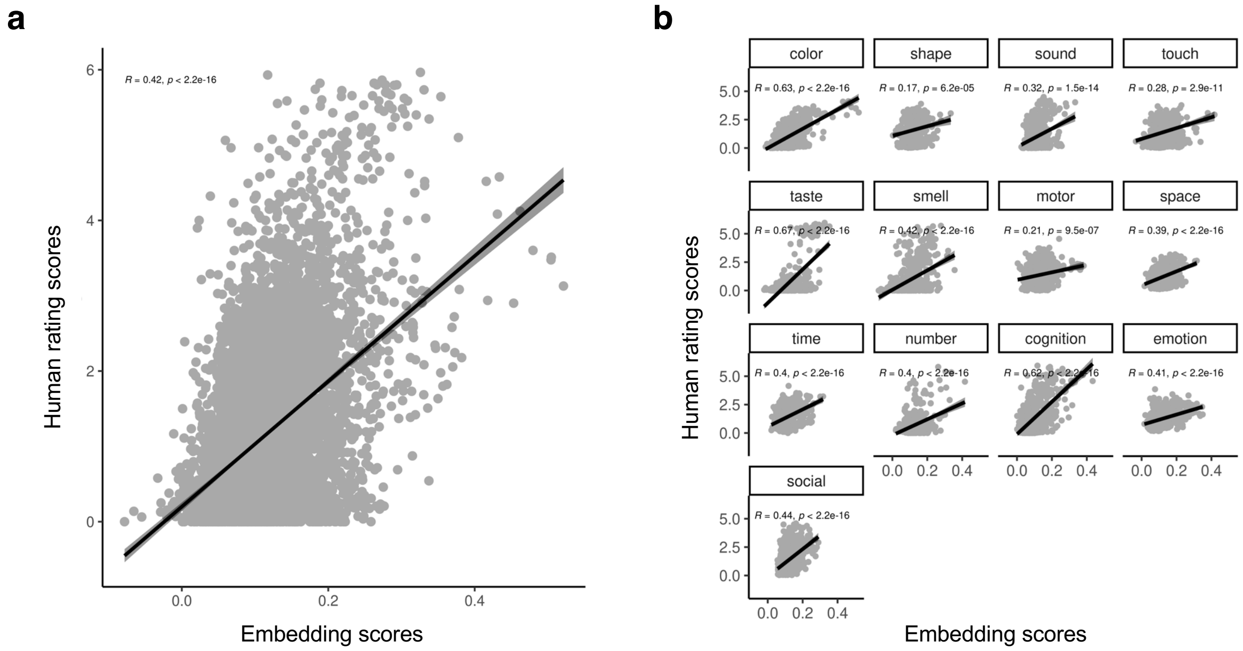

Figure S1. Validation of the language model as a measurement tool for probing neurocognitive semantic knowledge. We used a set of 535 English words and their human semantic ratings from Binder et al.[6] to examine the correlations between these ratings and word-dimension relations derived from the language model. **a**. The scatter plot of the 13-dimensional relations between human and language models, which demonstrate a moderate correlation (Pearson $r_{(6914)} = 0.42$, $P < 0.001$). **b**. Scatter plots of word-dimension relations for each semantic dimension. As some of our dimensions correspond to several ratings in Binder et al.[6], for these dimensions, the human rating data were first averaged and then correlated with our data derived from language models. The detailed correspondence between our dimensions and the averaged dimensions in Binder et al.[6] is shown as follows: shape-Shape, Large, Small, Pattern, and Complexity; color-Color, Dark, and Bright; touch-Touch, Temperature, Texture, Weight, and Pain; sound-Sound, Audition, Loud, Low, High, and Music; taste-Taste; smell-Smell; motor-Head, Upper limb, and Lower limb; space-Landmark, Path, Scene, Near, Toward, and Away; number-Number; time-Time, Duration, Long, Short, Caused, and Consequential; social-Social, Human, Communication, and Self; cognition-Cognition; emotion-Pleasant, Unpleasant, Happy, Sad, Angry, Disgusted, Fearful, and Surprised.

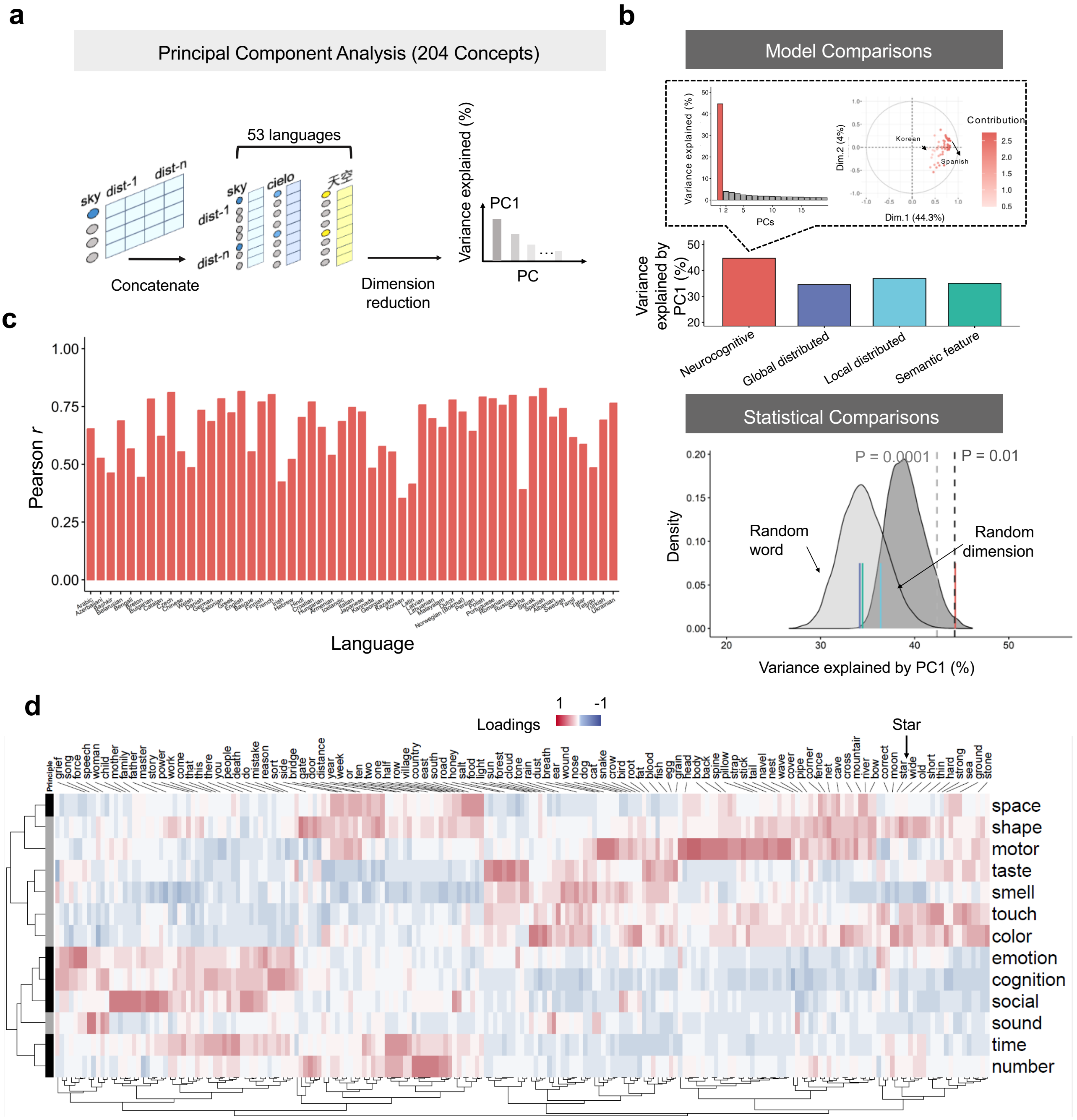

Figure S2. **Principal component results for 204 concepts shared across 53 languages. a.** Scheme of analyzing commonalities using principal component analysis. **b**. Principal component results. Upper panel: the bar plot shows the variance explained by PC1 for the four semantic models (N = 53), with the neurocognitive model exhibiting the highest variance explained. Above the bar plot is the scree plot and the distribution of languages along the first two PCs for the neurocognitive model. Lower panel: comparison of PC1-explained variance between the four semantic models and the two random control models. The neurocognitive model reached a significance of $P < 0.001$ in the random word distribution (light gray area) and of $P = 0.009$ in the random dimension distribution (dark gray area), while other models did not approach significance in either random control distribution. **c.** Correlation plots for all 53 languages with the first principal component of the neurocognitive semantic structure.

Positive correlations suggest that this component is present across all languages. **d.** Visualization of the PC1 neurocognitive semantic space: Shared loading patterns of 204 concepts on the 13 neurocognitive dimensions across 53 languages (red: high loading; blue: low loading). Dendrograms show the hierarchical clustering of concepts (columns) and of dimensions (rows; light gray: sensory-motor; black: social-cognitive) by correlation distances.

a

b

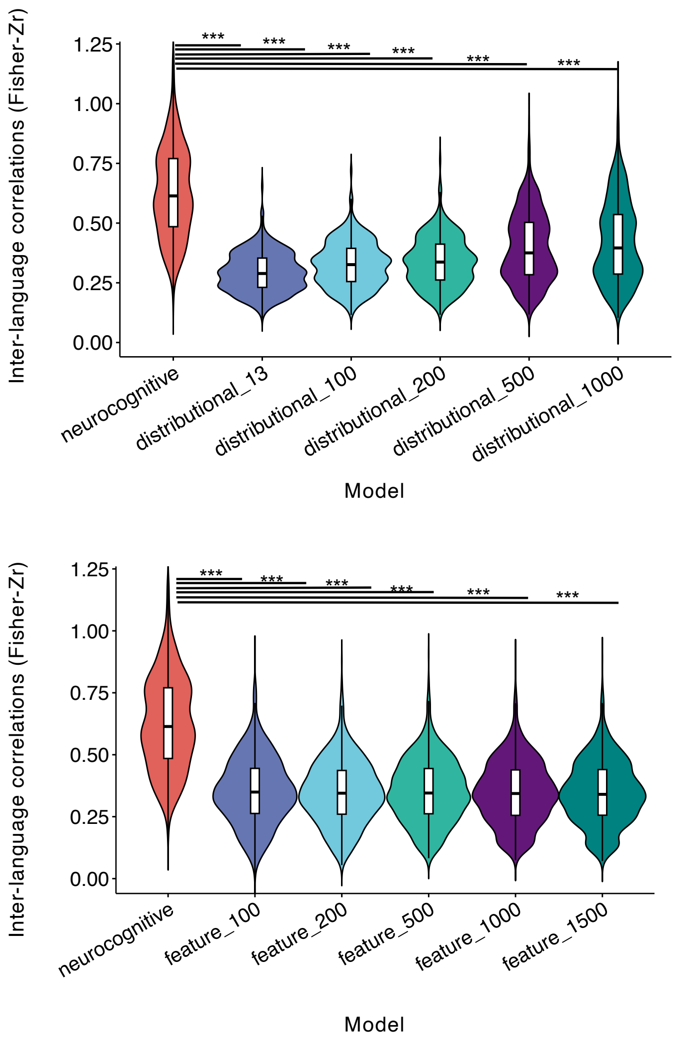

Figure S3. Validation of inter-language correlation results of distributional (local) and feature models with varying numbers of anchor words. In the main text, we compared the inter-language correlations of neurocognitive models with 100 anchor words in the distributional (local) model and in the feature model. Here, we validated the results using different numbers of anchor words. Violin plots show the distribution of inter-language correlations (Fisher-Z transformed) across 53 languages (N = 1,378 language pairs). White boxplots inside violins show the median (center line), 25th-75th percentiles (box limits), and whiskers extending to 1.5 × IQR. **a**. Distributional (local) model results. Concepts from the neurocognitive model exhibited stronger alignment than the distributional (local) models across anchor word counts ranging from 13 to 1,000 (all *P*s < 0.001). **b**. Feature model results. Concepts from the neurocognitive model demonstrated superior alignment compared to feature models across feature word counts spanning 100 to 1,000 (all *P*s < 0.001). ****P* < 0.001; two-sided paired Wilcoxon signed-rank test with FDR correction for multiple comparisons.

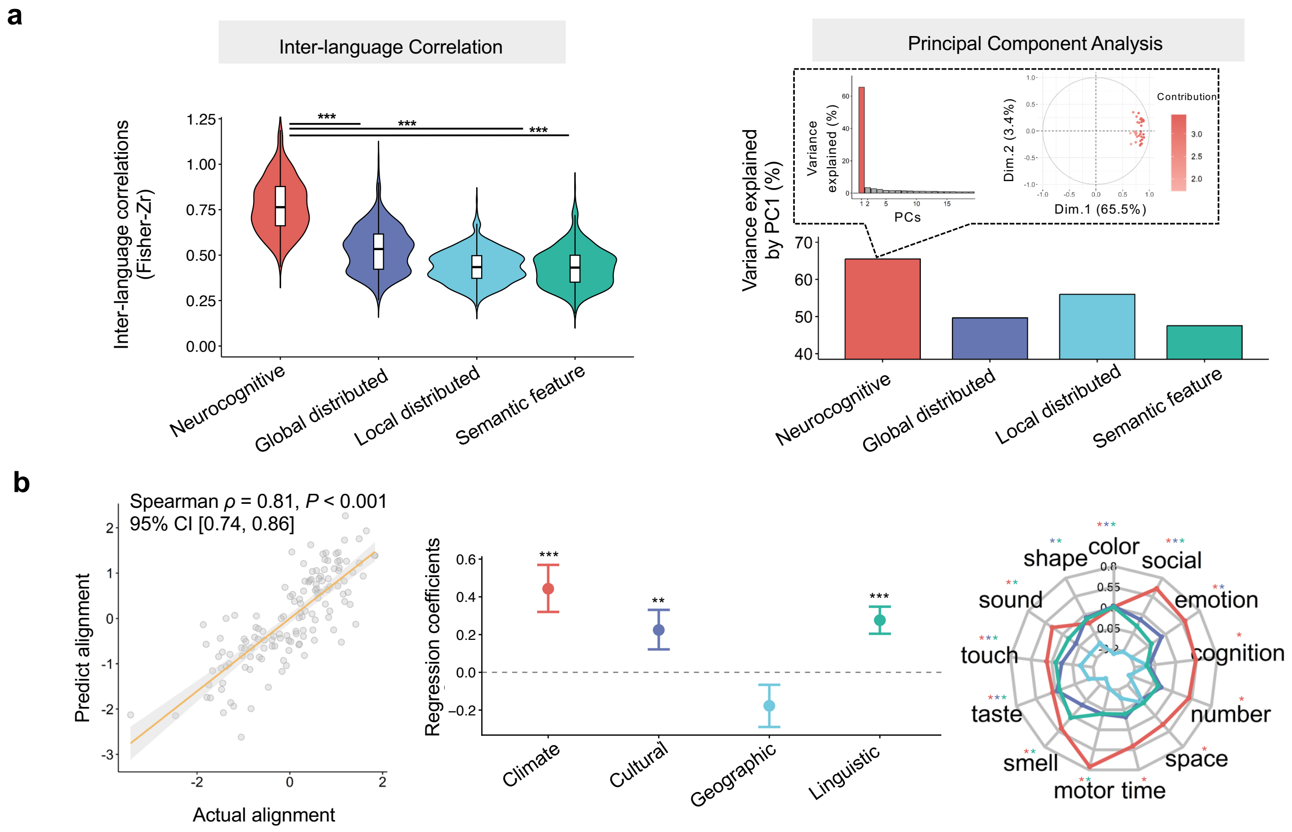

Figure S4. Validation of the main results using independent language models trained on the combination of Wikipedia and the Open Subtitle database. The figure represents validation analyses using word embedding models available for 35 languages[3], replicating main results on semantic commonalities and variation. **a.** Commonality analyses. Left panel: Violin plots show the distribution of the inter-language correlations (Fisher-Z transformed) across 35 languages (N = 595 language pairs). White boxplots inside violins show the median (center line), 25th-75th percentiles (box limits), and whiskers extending to 1.5 × IQR; two-sided paired Wilcoxon signed-rank test with FDR correction for multiple comparisons. Right panel: PCA results for 35 languages and 354 concepts. Variance explained by the first component (PC1) indicates higher commonalities in neurocognitive-based representation (65.62%) compared to other types of semantic structures (distributional--global: 45.28%; distributional-local: 55.94%; feature-based: 47.64%). Above the bar plot is the scree plot and the distribution of languages along the first two PCs for the neurocognitive model. **b**. Left panel: Scatter plot showing the relationship between actual and predicted semantic alignment values based on environmental variables (N = 17 languages, 136 unique language pairs). Each point represents a language pair. Shaded area represents 95% CI of the regression line. Middle panel: Standardized regression coefficients for each factor are shown as points. Right panel: Radar plot displaying standardized beta coefficients from regression models for each of the 13 neurocognitive dimensions across environmental variables. Error bars indicate 95% CIs. Two-sided tests: ***, $P < 0.001$, **, $P < 0.01$, *, $P < 0.05$.

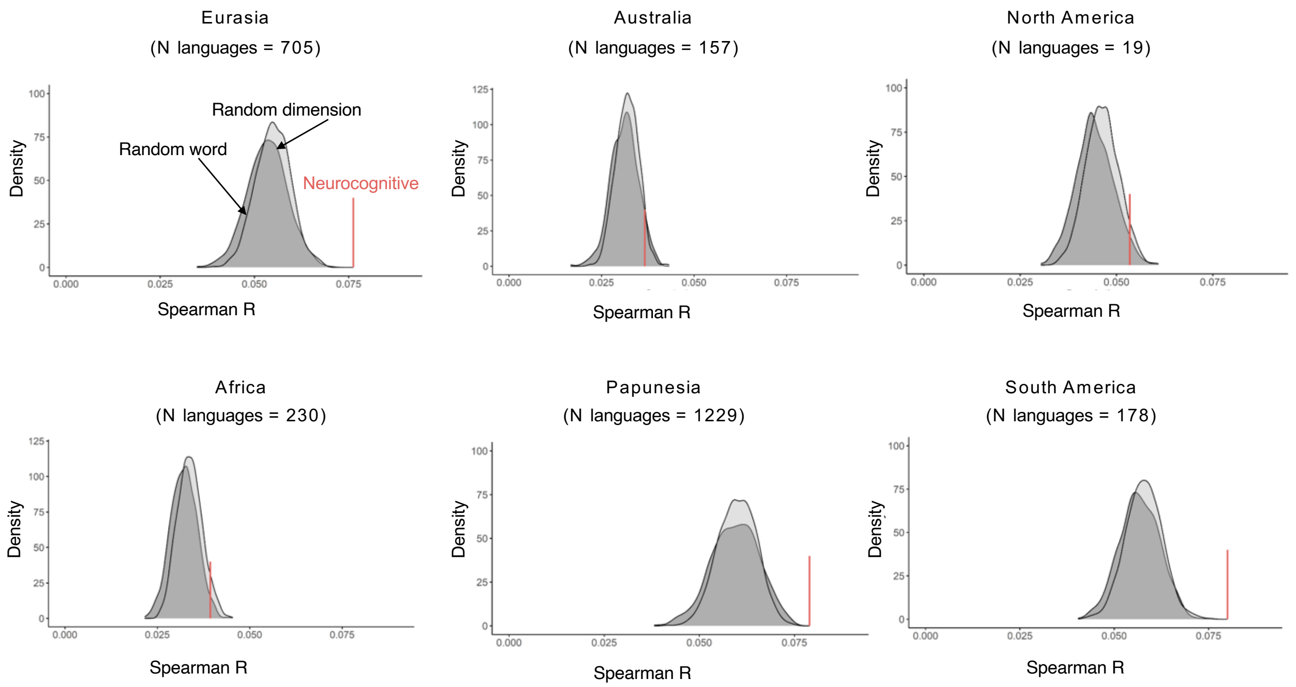

Figure S5. Generalization of 13-dimensional neurocognitive space to language samples in different geographic region areas using the colexification network topology. Density plots comparing association patterns between semantic distances in the original 53-language embedding-derived neurocognitive space and the colexification topological graph space. Analysis includes colexification networks from different language samples (Eurasia, Australia, North America, Africa, Papunesia, and South America). The neurocognitive model's performance is at the upper bound of the random model distributions.

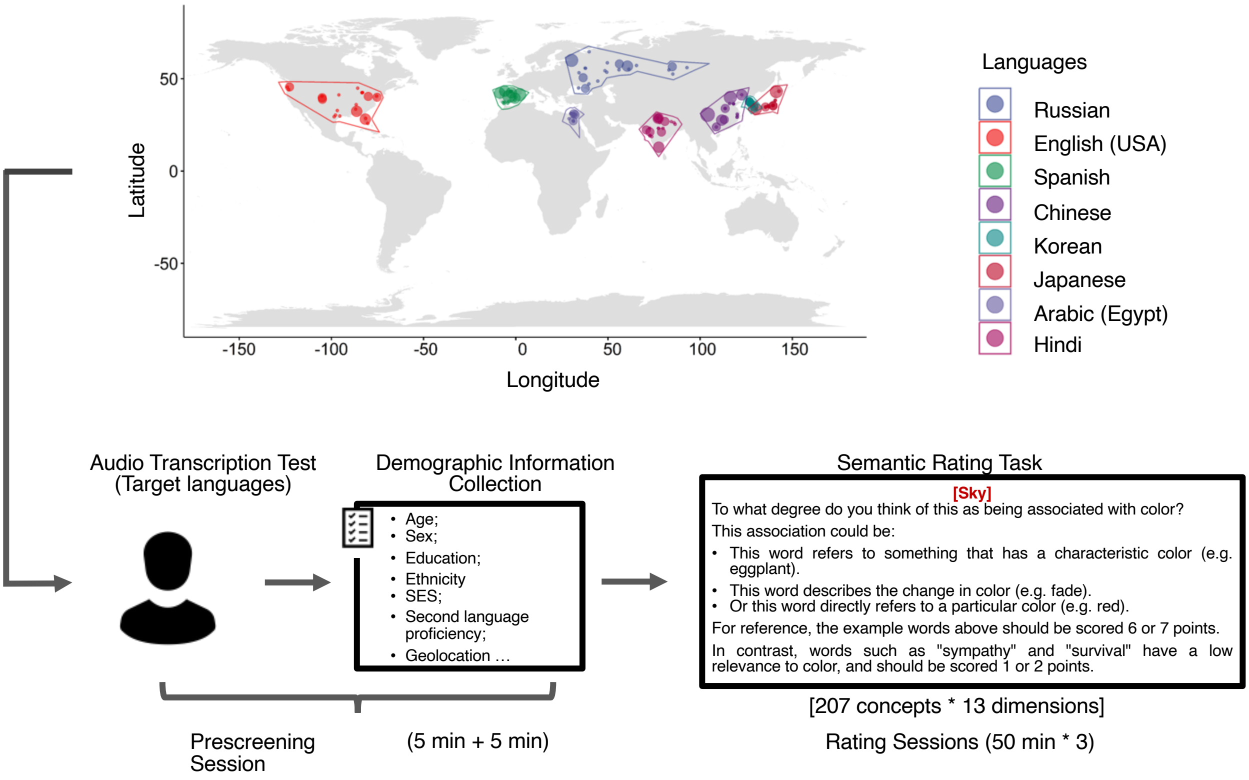

Figure S6. Sampling and data collection (Study 2). Diagrams outline the procedures for obtaining human ratings on 207 Swadesh concepts across 13 neurocognitive dimensions. We selected languages from Study 1, ensuring geographic, linguistic, and cultural diversity. Native speakers of 8 languages were recruited from 58 cities across 8 countries (Russia, USA, Spain, China, South Korea, Japan, Egypt, and India) via an online platform (http://www.appen.com). The participants completed a 5-minute audio transcription test in their native language and provided demographic information. After prescreening, they undertook the semantic rating task (over three sessions, each lasting approximately 50 minutes), associating concepts with each dimension.

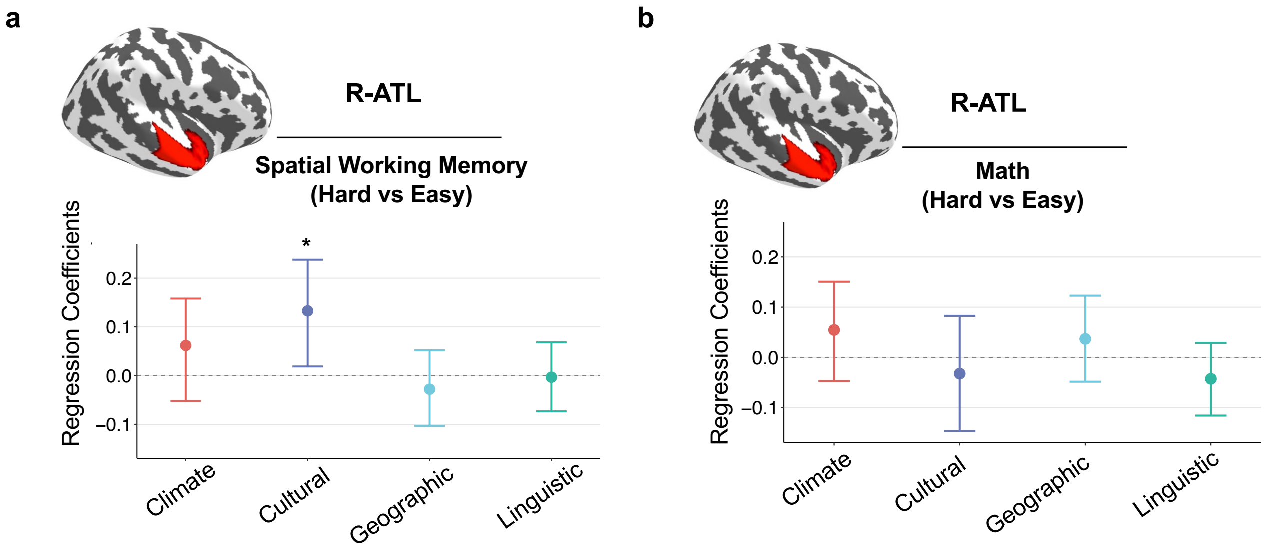

Figure S7. Beta coefficients from linear mixed-effects regression models predicting neural pattern dissimilarities in the right anterior temporal lobe (r-ATL) during nonlinguistic tasks (N = 33 languages, 528 independent language pairs). a. Spatial Working Memory task (Hard vs Easy condition), showing significant effects only for cultural distance ($\beta = 0.13$, $P = 0.016$, 95% CI [0.02, 0.24]) but not for climate distance ($\beta = 0.06$, $P = 0.213$, 95% CI [-0.05, 0.16]). **b**. Math task (Hard vs Easy condition), showing no significant effects for any environmental variable (all *P*s > 0.05). Error bars represent 95% CIs. *, $P < 0.05$.

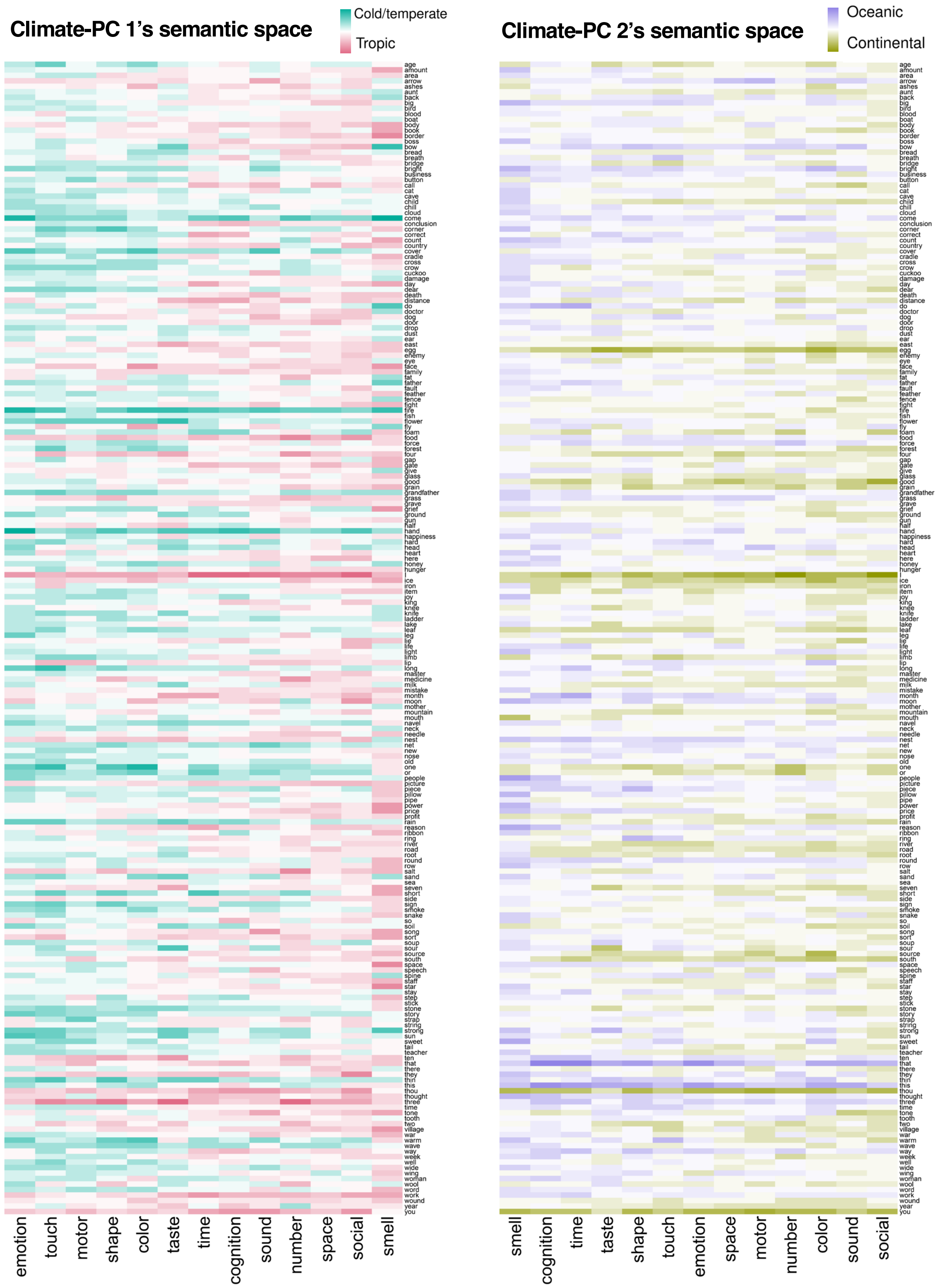

Figure S8. Semantic profiles associated with Climate PC1 and Climate PC2 at the concept level. Higher values along a particular direction of PCs indicate that the given climate type tends to have stronger semantic relations.

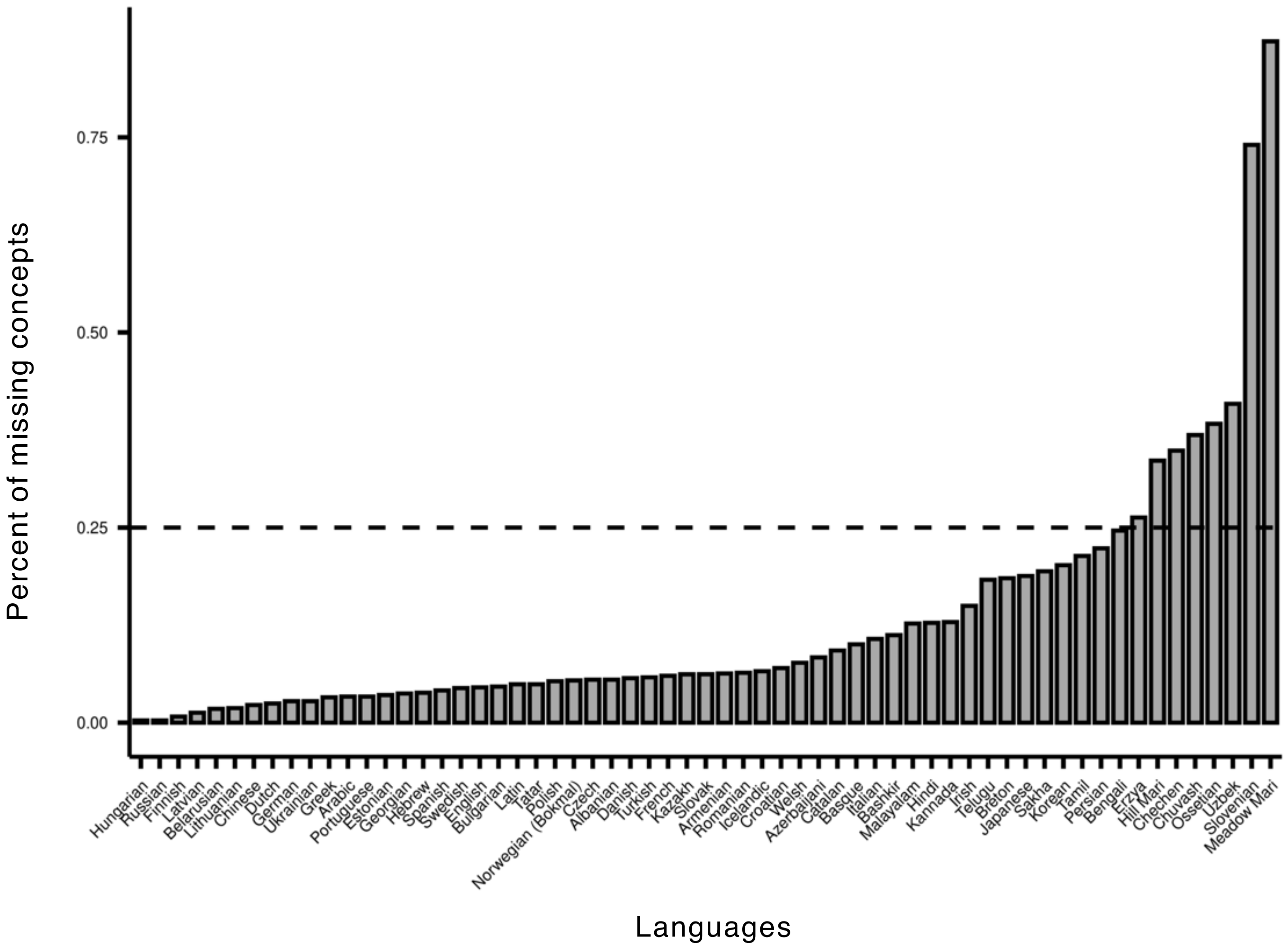

Figure S9. Distribution of NEL concept omission in language computation models for 61 candidate languages. Eight languages were excluded from main analyses due to >25% missing concept representations, including Erzya, Hill Mari, Chechen, Chuvash, Ossetian, Uzbek, Slovenian, and Meadow Mari.

Table S1. Example (non-exhaustive) empirical references contributing to the neurocognitive dimensional framework (gleaned from representative reviews and meta-analyses studies of the neural basis of semantic processing)**.**

Dimensions	Task	Stimuli	Language	Contrast	Imaging modality	Year	References
Color	Word generation	pictures, words	NA	Color > Action	PET	1995	Martin, A., Haxby, J. V., Lalonde, F. M., Wiggs, C. L., & Ungerleider, L. G. (1995). Discrete cortical regions associated with knowledge of color and knowledge of action. Science, 270(5233), 102-105.
	Semantic decision	words	English	Color > Non-color (Achromatic)	fMRI	2012	Hsu, N. S., Frankland, S. M., & Thompson-Schill, S. L. (2012). Chromaticity of color perception and object color knowledge. Neuropsychologia, 50(2), 327-333.
	Semantic decision	words	Italian	Color > Action	fMRI	2020	Bottini, R., Ferraro, S., Nigri, A., Cuccarini, V., Bruzzone, M. G., & Collignon, O. (2020). Brain regions involved in conceptual retrieval in sighted and blind people. Journal of Cognitive Neuroscience, 32(6), 1009-1025.
Shape	Seman decision	words	English	Shape > (Action, Sound)	fMRI	2003	Noppeney, U., Friston, K. J., & Price, C. J. (2003). Effects of visual deprivation on the organization of the semantic system. Brain, 126(7), 1620-1627.

	Lexical decision	words	English	Visual > Sound	fMRI	2013	Bonner, M. F., Peelle, J. E., Cook, P. A., & Grossman, M. (2013). Heteromodal conceptual processing in the angular gyrus. NeuroImage, 71, 175-186.
	Semantic decision	words	Chinese	Shape RSA effects	fMRI	2014	Peelen, M. V., He, C., Han, Z., Caramazza, A., & Bi, Y. (2014). Nonvisual and visual object shape representations in occipitotemporal cortex: evidence from congenitally blind and sighted adults. Journal of Neuroscience, 34(1), 163-170.
Sound	Semantic decision	words	English	Sound > (Taste, Touch, Color)	fMRI	2006	Goldberg, R. F., Perfetti, C. A., & Schneider, W. (2006). Perceptual knowledge retrieval activates sensory brain regions. Journal of Neuroscience, 26(18), 4917-4921.
	Lexical decision	words	English	Sound > Visual	fMRI	2013	Bonner, M. F., Peelle, J. E., Cook, P. A., & Grossman, M. (2013). Heteromodal conceptual processing in the angular gyrus. NeuroImage, 71, 175-186.
	Lexical decision	words	German	Sound > Non-sound	fMRI	2008	Kiefer, M., Sim, E. J., Herrnberger, B., Grothe, J., & Hoenig, K. (2008). The sound of concepts: Four markers for a link between auditory and conceptual brain systems. Journal of Neuroscience, 28(47), 12224-12230.

Touch	Semantic decision	words	English	Touch > (Sound, Taste, Color)	fMRI	2006	Goldberg, R. F., Perfetti, C. A., & Schneider, W. (2006). Perceptual knowledge retrieval activates sensory brain regions. Journal of Neuroscience, 26(18), 4917-4921.
	Mental imagery	pictures	NA	Hand > Body	fMRI	2016	Perruchoud, D., Michels, L., Piccirelli, M., Gassert, R., & Ionta, S. (2016). Differential neural encoding of sensorimotor and visual body representations. Scientific Reports, 6(1), 37259.
Taste	Semantic decision	words	English	Taste > (Sound, Touch, Color)	fMRI	2006	Goldberg, R. F., Perfetti, C. A., & Schneider, W. (2006). Perceptual knowledge retrieval activates sensory brain regions. Journal of Neuroscience, 26(18), 4917-4921.
	Passive reading	words	Spanish	Taste > Non-taste	fMRI	2012	Barros-Loscertales, A., González, J., Pulvermüller, F., Ventura-Campos, N., Bustamante, J. C., Costumero, V., ... & Ávila, C. (2012). Reading salt activates gustatory brain regions: fMRI evidence for semantic grounding in a novel sensory modality. Cerebral Cortex, 22(11), 2554-2563.
	Semantic decision	words	Italian	Food > (People, Place)	fMRI	2020	Fairhall, S. L. (2020). Cross recruitment of domain-selective cortical representations enables flexible semantic knowledge. Journal of Neuroscience, 40(15), 3096-3103.

Smell	Passive reading	words	Spanish	Smell > Non-smell	fMRI	2006	González, J., Barros-Loscertales, A., Pulvermüller, F., Meseguer, V., Sanjuán, A., Belloch, V., & Ávila, C. (2006). Reading cinnamon activates olfactory brain regions. NeuroImage, 32(2), 906-912.
	Semantic decision	sentences	German	Smell > Non-smell	fMRI	2018	Pomp, J., Bestgen, A. K., Schulze, P., Müller, C. J., Citron, F. M., Suchan, B., & Kuchinke, L. (2018). Lexical olfaction recruits olfactory orbitofrontal cortex in metaphorical and literal contexts. Brain and Language, 179, 11-21.
Motor	Passive reading	words	English	Action > #	fMRI	2004	Hauk, O., Johnsrude, I., & Pulvermüller, F. (2004). Somatotopic representation of action words in human motor and premotor cortex. Neuron, 41(2), 301-307.
	Word generation	pictures, words	NA	Action > Color	PET	1995	Martin, A., Haxby, J. V., Lalonde, F. M., Wiggs, C. L., & Ungerleider, L. G. (1995). Discrete cortical regions associated with knowledge of color and knowledge of action. Science, 270(5233), 102-105.

	Semantic decision	passage	English	Action > (Percept, Emotion)	fMRI	2014	Chow, H. M., Mar, R. A., Xu, Y., Liu, S., Wagage, S., & Braun, A. R. (2014). Embodied comprehension of stories: interactions between language regions and modality-specific neural systems. Journal of Cognitive Neuroscience, 26(2), 279-295.
Time	Sentence judgment	sentences	English	Time > Non-time	fMRI	2016	Lai, V. T., & Desai, R. H. (2016). The grounding of temporal metaphors. Cortex, 76, 43-50.
Space	Semantic decision	words	NA	Space > Color	PET	1998	Mummery, C. J., Patterson, K., Hodges, J. R., & Price, C. J. (1998). Functional neuroanatomy of the semantic system: divisible by what? Journal of Cognitive Neuroscience, 10(6), 766-777.
	Naming	pictures	English	Space > Object	PET	2001	Damasio, H., Grabowski, T. J., Tranel, D., Ponto, L. L., Hichwa, R. D., & Damasio, A. R. (2001). Neural correlates of naming actions and of naming spatial relations. NeuroImage, 13(6), 1053-1064.

	Semantic decision	pictures, words	Italian	Place > People	fMRI	2014	Fairhall, S. L., Anzellotti, S., Ubaldi, S., & Caramazza, A. (2014). Person-and place-selective neural substrates for entity-specific semantic access. Cerebral Cortex, 24(7), 1687-1696.
Number	Semantic decision	words	NA	Number > Animal	PET	2005	Thioux, M., Pesenti, M., Costes, N., De Volder, A., & Seron, X. (2005). Task-independent semantic activation for numbers and animals. Cognitive Brain Research, 24(2), 284-290.
	Sentence comprehension	sentences	English	Number > Non-number	fMRI	2005	McMillan, C. T., Clark, R., Moore, P., Devita, C., & Grossman, M. (2005). Neural basis for generalized quantifier comprehension. Neuropsychologia, 43(12), 1729-1737.
Emotion	Perceptual decision	words	NA	Threat > Neutral	PET	1999	Isenberg, N., Silbersweig, D., Engelien, A., Emmerich, S., Malavade, K., Beattie, B., ... & Stern, E. (1999). Linguistic threat activates the human amygdala. Proceedings of the National Academy of Sciences, 96(18), 10456-10459.
	Lexical decision	words	English	Negative > Neural	fMRI	2006	Nakic, M., Smith, B. W., Busis, S., Vythilingam, M., & Blair, R. J. R. (2006). The impact of affect and frequency on lexical decision: the role of the amygdala and inferior frontal cortex. NeuroImage, 31(4), 1752-1761.

	Semantic decision	words	English	Emotion > Non-emotion	fMRI	2019	Wang, X., Wang, B., & Bi, Y. (2019). Close yet independent: Dissociation of social from valence and abstract semantic dimensions in the left anterior temporal lobe. Human Brain Mapping, 40(16), 4759-4776.
Cognition	Sentence comprehension	sentences	English	Mental > Action	fMRI	2012	Kana, R. K., Blum, E. R., Ladden, S. L., & Ver Hoef, L. W. (2012). "How to do things with Words": Role of motor cortex in semantic representation of action words. Neuropsychologia, 50(14), 3403-3409.
	Mental imagery	words	Italian	Mental > Physical	fMRI	2014	Tomasino, B., Fabbro, F., & Brambilla, P. (2014). How do conceptual representations interact with processing demands: An fMRI study on action- and abstract-related words. Brain Research, 1591, 38-52.
Social	Story comprehension	passages	English	Social > Physical	PET	1995	Fletcher, P. C., Happe, F., Frith, U., Baker, S. C., Dolan, R. J., Frackowiak, R. S., & Frith, C. D. (1995). Other minds in the brain: a functional imaging study of "theory of mind" in story comprehension. Cognition, 57(2), 109-128.
	Semantic decision	words	English	Social > Non-social	fMRI	2012	Contreras, J. M., Banaji, M. R., & Mitchell, J. P. (2012). Dissociable neural correlates of stereotypes and other forms of semantic knowledge. Social Cognitive and Affective Neuroscience, 7(7), 764-770.

	Semantic decision	words	Chinese	Social > Non-social	fMRI	2019	Lin, N., Xu, Y., Wang, X., Yang, H., Du, M., Hua, H., & Li, X. (2019). Coin, telephone, and handcuffs: Neural correlates of social knowledge of inanimate objects. Neuropsychologia, 133, 107187.

Table S2. Language samples included across three studies

Language	ISO3*	Language family	Study
Afrikaans	afr	Indo-European	Study 3
Albanian	sqi	Indo-European	Study 1
Arabic	arb	Afro-Asiatic	Study 1, Study 2, Study 3
Armenian	hye	Indo-European	Study 1, Study 3
Azerbaijani	azj	Turkic	Study 1
Bashkir	bak	Turkic	Study 1
Basque	eus	Basque	Study 1, Study 3
Belarusian	bel	Indo-European	Study 1, Study 3
Bengali	ben	Indo-European	Study 1
Breton	bre	Indo-European	Study 1
Bulgarian	bul	Indo-European	Study 1, Study 3
Catalan	cat	Indo-European	Study 1, Study 3
Chinese	cmn	Sino-Tibetan	Study 1, Study 2, Study 3
Croatian	hrv	Indo-European	Study 1, Study 3
Czech	ces	Indo-European	Study 1, Study 3
Danish	dan	Indo-European	Study 1, Study 3
Dutch	nld	Indo-European	Study 1, Study 3
English	eng	Indo-European	Study 1, Study 2, Study 3
Estonian	ekk	Uralic	Study 1
Farsi	pes	Indo-European	Study 3
Finnish	fin	Uralic	Study 1, Study 3
French	fra	Indo-European	Study 1, Study 3
Georgian	kat	Kartvelian	Study 1
German	deu	Indo-European	Study 1, Study 3
Greek	ell	Indo-European	Study 1, Study 3
Gujarati	guj	Indo-European	Study 3
Hebrew	heb	Afro-Asiatic	Study 1, Study 3
Hindi	hin	Indo-European	Study 1, Study 2, Study 3
Hungarian	hun	Uralic	Study 1, Study 3
Icelandic	isl	Indo-European	Study 1
Irish	gle	Indo-European	Study 1, Study 3
Italian	ita	Indo-European	Study 1, Study 3
Japanese	jpn	Japonic	Study 1, Study 2, Study 3
Kannada	kan	Dravidian	Study 1
Kazakh	kaz	Turkic	Study 1
Korean	kor	Koreanic	Study 1, Study 2, Study 3
Latin	lat	Indo-European	Study 1
Latvian	lav	Indo-European	Study 1, Study 3
Lithuanian	lit	Indo-European	Study 1, Study 3
Malayalam	mal	Dravidian	Study 1
Marathi	mar	Indo-European	Study 3
Nepali	npi	Indo-European	Study 3

Norwegian (Bokmal)	nor	Indo-European	Study 1, Study 3
Persian	pes	Indo-European	Study 1
Polish	pol	Indo-European	Study 1, Study 3
Portuguese	por	Indo-European	Study 1, Study 3
Romanian	ron	Indo-European	Study 1, Study 3
Russian	rus	Indo-European	Study 1, Study 2, Study 3
Sakha	sah	Turkic	Study 1
Slovak	slk	Indo-European	Study 1
Slovene	slv	Indo-European	Study 3
Spanish	spa	Indo-European	Study 1, Study 2, Study 3
Swahili	swh	Niger-Congo	Study 3
Swedish	swe	Indo-European	Study 1, Study 3
Tagalog	tgl	Austroasiatic	Study 3
Tamil	tam	Dravidian	Study 1, Study 3
Tatar	tat	Turkic	Study 1
Telugu	tel	Dravidian	Study 1, Study 3
Turkish	tur	Turkic	Study 1, Study 3
Ukrainian	ukr	Indo-European	Study 1, Study 3
Vietnamese	vie	Austroasiatic	Study 3
Welsh	cym	Indo-European	Study 1

* Notes: International Organization for Standardization (ISO) codes.

Table S3. Anchor words used to probe the neurocognitive semantic structure

Anchor words	Concept ID (in NEL)	Neurocognitive dimensions
eye	Auge::N	motor
ear	Ohr::N	
nose	Nase::N	
mouth	Mund::N	
tooth	Zahn::N	
tongue	Zunge::N	
shoulder	Schulter::N	
arm	Arm::N	
elbow	Ellenbogen::N	
hand	Hand::N	
finger	Finger::N	
foot	Fuß::N	
leg	Bein::N	
body	Körper::N	
bright	hell::A	color
dark	dunkel::A	
black	schwarz::A	
white	weiß::A	
red	rot::A	
yellow	gelb::A	
blue	blau::A	
green	grün::A	
grey	grau::A	
shine	scheinen[Sonne]::V	
colourful	bunt::A	
hard	hart::A	touch
soft	weich::A	
heavy	schwer::A	
hot	heiß::A	
warm	warm::A	
cold	kalt::A	
damp	feucht::A	
wet	nass::A	
dry	trocken::A	
sharp	scharf::A	
blunt	stumpf::A	
sweet	süß::A	taste
bitter	bitter::A	
flavour	Geschmack::N	
salt	Salz::N	
sour	sauer::A	
odour	Geruch::N	smell

smell	riechen::V	
noise	Lärm::N	sound
sound	Laut::N	
tone	Ton::N	
hear	vernehmen::V	
listen	zuhören::V	
calm	Ruhe::N	
circle	Kreis::N	shape
line	Linie::N	
size	Größe::N	
long	lang::A	
short	kurz::A	
big	groß::A	
little	klein::A	
round	rund::A	
space	Platz::N	space
place	Ort::N	
side	Seite::N	
middle	Mitte::N	
edge	Kante::N	
near	nah::A	
far	fern::A	
left	linker::A	
right	rechter::A	
down	hinab::ADV	
up	hinauf::ADV	
ahead	geradeaus::ADV	
behind	hinter::PRP	
end	Schluss::N	time
time	Zeit::N	
old	alt::A	
new	neu::A	
long	lange::ADV	
suddenly	plötzlich::ADV	
instantly	sofort::ADV	
later	später::ADV	
now	jetzt::ADV	
soon	bald::ADV	
before	vorher::ADV	
afterwards	danach::ADV	
end	enden::V	
begin	beginnen::V	
full	voll::A	number
empty	leer::A	

half	Hälfte::N	
one	eins::NUM	
two	zwei::NUM	
three	drei::NUM	
amount	Menge::N	
memory	Gedächtnis::N	cognition
mind	Verstand::N	
think	denken::V	
believe	glauben::V	
understand	verstehen::V	
know	wissen::V	
remember	sich erinnern an::V	
forget	vergessen::V	
hence	von hier::ADV	
reason	Grund::N	
good	gut::A	emotion
bad	schlecht::A	
want	wollen::V	
love	lieben::V	
happiness	Glück::N	
grief	Kummer::N	
afraid	fürchten::V	
annoy	ärgern::V	
sad	traurig::A	
joy	Freude::N	
desire	Lust::N	
merry	lustig::A	
human	Mensch::N	social
man	Mann::N	
woman	Frau::N	
child	Kind::N	
family	Familie::N	
parents	Eltern::N	
people	Leute::N	
nation	Volk::N	
help	Hilfe::N	
friend	Freund::N	
enemy	Feind::N	
I	ich::PRN	
we	wir::PRN	
you	ihr::PRN	
they	sie::PRN	

Table S4. Correlational matrix between environmental variables and semantic variations (Study 1)

	Climate	Linguistic history	Geography	Culture	Semantic
Climate	-	-	-	-	-
Linguistic history	0.48 [0.44, 0.52]	-	-	-	-
Geography	0.79 [0.76, 0.80]	0.67 [0.63, 0.70]	-	-	-
Culture	0.73 [0.68, 0.78]	0.37 [0.28, 0.45]	0.52 [0.44, 0.58]	-	-
Semantic	0.53 [0.49, 0.58]	0.39 [0.34, 0.44]	0.45 [0.41, 0.49]	0.49 [0.41, 0.57]	-

Note. All correlations are Spearman's ρ based on N = 1,378 unique language pairs (53 languages). Values in brackets represent 95% confidence intervals computed via percentile bootstrap (10,000 iterations). All *P*s < 0.001.

Table S5. Geographic sampling of participants in Study 2

Country	City	Number of participants
Russia	Volga Federal District	6
	South Federal District	5
	Central Federal District	5
	Urals Federal District	5
	Siberian Federal District	5
	North West Federal District	5
USA	Pennsylvania	5
	Florida	5
	Colorado	5
	Alabama	4
	Michigan	4
	Texas	4
	Oregon	4
	Tennessee	1
	Nebraska	1
Spain	Andalusia	5
	Valencian Community	5
	Community of Madria	4
	Catalonia	4
	Asturias	3
	Basque Country	3
	Extremadura	3
	Galicia	3
China	Sichuan	5
	Hunan	5
	Zhejiang	4
	Henan	4
	Shandong	4
	Liaoning	4
	Beijing	4
	Guangxi	3
South Korea	Busan	6
	Seoul	5
	Incheon	5
	Gyeonggi-do	5
	Gyeongsangnam-do	4
	Daejeon	3
	Daegu	3
Japan	Tokyo	5
	Hokoaido	5
	Aichi	5
	Kanagawa	5

	Osaka	5
	Fukuoka	5
	Miyagi Prefecture	1
Egypt	Cairo	8
	Dakahlia	6
	Alexandria	5
	Ismailia	5
	Giza	5
	Asyut	5
India	Delhi	6
	Maharashtra	5
	Gujarat	5
	Madhya Pradesh	4
	Uttar Pradesh	4
	Bihar	3
	Karnataka	3

Table S6. Word lists of Swadesh 207 concepts in 8 languages

No.	English (en)	Chinese (cn)	Arabic (ar)	Hindi (hi)	Japanese (ja)	Korean (ko)	Russian (ru)	Spanish (es)
1	I	我	أنا	मैं	私	나	я	yo
2	you (singular)	你	أنت	तुम	あなた	너	ты	tú
3	he	他	هو	वह	彼	그	он	él
4	we	我们	نحن	हम	私たち	우리	мы	nosotros
5	you (plural)	你们	أنتم	आप	あなた達	너희들	вы	vosotros
6	they	他们	هم	वे	彼ら	그들	они	ellos
7	this	这个	هذا	यह	この	이-	это	este
8	that	那个	ذلك	वह	あの	그-	то	aquel
9	here	这里	هنا	यहाँ	ここ	이곳	тут	aquí
10	there	那里	هناك	वहाँ	あそこ	그곳	там	allí
11	who	谁	من	कौन	誰	누구	кто	quien
12	what	什么	ما	क्या	何	무엇	что	que
13	where	哪里	أين	कहाँ	どこ	어디	где	donde
14	when	什么时候	متى	कब	いつ	언제	когда	cuando
15	how	怎么	كيف	कैसे	どう	어떻게	как	como
16	not	不是	لا	नहीं	無い	-아니다	не	no
17	all	全部	كل	सभी	すべて	모든	всё (совокупность чего-либо)	todos
18	many	多	كثير	बहुत	多い	많은	много	muchos
19	some	一些	بعض	कुछ	幾つか	어떤	несколько	algunos
20	few	少	قليل	थोड़े	少し	조금	мало	poco
21	other	另外的	أخرى	दूसरा	他	다른	другой	otro
22	one	一	واحد	एक	一	하나	один	uno
23	two	二	اثنان	दो	二	둘	два	dos
24	three	三	ثلاثة	तीन	三	셋	три	tres

25	four	四	أربعة	चार	四	넷	четыре	cuatro
26	five	五	خمسة	पाँच	五	다섯	пять	cinco
27	big	大	كبير	बड़ा	大きい	크다	большой (обладающий немаленьким размером)	grande
28	long	长	طويل	लम्बा	長い	길다	длинный (большой по протяженности или длительности)	largo
29	wide	宽	عريض	चौड़ा	広い	넓다	широкий	ancho
30	thick	厚	سميك	मोटा	厚い	두껍다	толстый	grueso
31	heavy	重	ثقيل	भारी	重い	무겁다	тяжелый (обладающий большим весом)	pesado
32	small	小	صغير	छोटा	小さい	작다	маленький	pequeño
33	short	短	قصير	लघु	短い	짧다	короткий	corto
34	narrow	窄	ضيق	तंग	狭い	좁다	узкий	estrecho
35	thin	细	رفيع	पतला	細い	얇다	тонкий	delgado
36	woman	女人	امرأة	औरत	女	여자	женщина	mujer
37	man (as adult male)	男人	رجل	पुरुष	男	남자	мужчина	hombre
38	man (as human being)	人	إنسان	इंसान	人	사람	человек	humano
39	child	儿童	طفل	बच्चा	子供	어린이	ребенок	niño
40	wife	妻子	زوجة	पत्नी	妻	아내	жена	esposa
41	husband	丈夫	زوج	पति	夫	남편	муж	esposo
42	mother	妈妈	أم	माँ	母	어머니	мать	madre
43	father	爸爸	أب	पिता	父	아버지	отец	padre
44	animal	动物	حيوان	जानवर	動物	동물	животное	animal

45	fish	鱼	سمك	मछली	魚	물고기	рыба	pez
46	bird	鸟	طائر	पक्षी	鳥	새	птица	pájaro
47	dog	狗	كلب	कुत्ता	犬	개	собака	perro
48	louse	虱子	قملة	जूँ	シラミ	이 (곤충)	вошь	piojo
49	snake	蛇	أفعى	साँप	蛇	뱀	змея	serpiente
50	worm	蚯蚓	دودة	कृमि	虫	벌레	червь	gusano
51	tree	树	شجرة	पेड़	木	나무	дерево	árbol
52	forest	森林	غابة	वन	林	숲	лес	bosque
53	branch	树枝	غصن	शाखा	枝	나뭇가지	ветка (часть дерева)	rama
54	fruit	水果	فاكهة	फल	果物	과일	фрукт (плод)	fruto
55	seed	种子	بذرة	बीज	種	씨	семя	semilla
56	leaf	叶子	ورقة	पत्ती	葉	잎	Лист (часть растения)	hoja
57	root	根	جذر	जड़	根	뿌리	корень (подземная часть дерева)	raíz
58	bark (of a tree)	树皮	لحاء	(पेड़ की) छाल	樹皮	나무껍질	кора	corteza
59	flower	花	زهرة	पुष्प	花	꽃	цветок	flor
60	grass	草	حشيش	घास	草	풀	трава	hierba
61	rope	绳	حبل	रस्सी	ロープ	밧줄	верёвка	cuerda
62	skin	皮肤	جلد	त्वचा	皮	피부	кожа	piel
63	meat	肉	لحم	गोश्त	肉	고기	мясо	carne
64	blood	血	دم	रक्त	血	피	кровь	sangre
65	bone	骨头	عظم	हड्डी	骨	뼈	кость	hueso
66	fat (noun)	脂肪	دهن	वसा	油	기름	жир	grasa
67	egg	蛋	بيضة	अंडा	卵	알	яйцо	huevo
68	horn	角	قرن	सींग; (भोंपू)	角	뿔	рог	cuerno

69	tail	尾巴	ذيل	पूँछ	尻尾	꼬리	хвост	cola
70	feather	羽毛	ريشة	मुलायम पंख	羽	깃털	перо	pluma
71	hair	头发	شعر	केश	毛	머리카락	волос	pelo
72	head	头	رأس	सिर	頭	머리	голова	cabeza
73	ear	耳朵	أذن	कान	耳	귀	ухо	oreja
74	eye	眼睛	عين	आँख	目	눈 (기관)	глаз	ojo
75	nose	鼻子	أنف	नाक	鼻	코	нос	nariz
76	mouth	嘴	فم	मुँह	口	입	рот	boca
77	tooth	牙	سن	दाँत	歯	이 (기관)	зуб	diente
78	tongue (organ)	舌头	لسان	जीभ	舌	혀	язык	lengua
79	fingernail	指甲	ظفر	नाखून	爪	손톱	ноготь	uña
80	foot	脚	قدم	पैर	足	발	стопа	pie
81	leg	腿	ساق	टांग	脚	다리	нога	pierna
82	knee	膝盖	ركبة	घुटना	膝	무릎	колено	rodilla
83	hand	手	يد	हाथ	手	손	рука	mano
84	wing	翅膀	جناح	पंख	翼	날개	крыло	ala
85	belly	肚子	بطن	पेट	腹	배	живот	barriga
86	guts	内脏	أمعاء	अंतड़ी	内臓	내장	кишечник	intestinos
87	neck	脖子	عنق	गर्दन	首	목	шея	cuello
88	back	背	ظهر	पीठ, (पीछे)	背	등	спина	espalda
89	breast	胸	صدر	छाती	胸	가슴	грудь	pecho
90	heart	心	قلب	दिल	心臓	심장	сердце	corazón
91	liver	肝	كبد	कलेजा	肝臓	간	печень	hígado
92	drink	喝	شرب	पीना, (पेय पदार्थ)	飲む	마시다	пить	beber
93	eat	吃	أكل	खाना	食べる	먹다	есть	comer
94	bite	咬	عضّ	मुँह से काटना	噛む	물다	кусать	morder
95	suck	吮吸	امتصّ	चूसना	吸う	빨다	сосать	chupar

96	spit	吐	بصق	थूकना	唾を吐く	침뱉다	плевать	escupir
97	vomit	呕吐	تقيأ	उल्टी करना	吐く	토하다	тошнить	vomitar
98	blow	吹	نفخ	फूँकना	吹く	불다	дуть	soplar
99	breathe	呼吸	تنفس	साँस लेना	呼吸する	숨쉬다	дышать	respirar
100	laugh	笑	ضحك	हँसना	笑う	웃다	смеяться	reír
101	see	看	رأى	देखना	見る	보다	видеть	ver
102	hear	听	سمع	सुनना	聞く	듣다	слышать	oír
103	know	知道	علم	जानना	知る	알다	знать	saber
104	think	思考	افتكر	सोचना	思う	생각하다	думать	pensar
105	smell	闻	اشتم	सूँघना	匂う	냄새	нюхать	oler
106	fear	害怕	خاف	डरना	恐れる	두려움	бояться	temer
107	sleep	睡觉	نام	सोना	寝る	자다	спать	dormir
108	live	活	عاش	जीवित रहना	生きている	살다	жить	vivir
109	die	死	مات	मरना	死ぬ	죽다	умирать	morir
110	kill	杀	قتل	जान से मारना	殺す	죽이다	убивать	matar
111	fight	打架	تقاتل	लड़ना	争う	싸우다	драться	luchar
112	hunt	打猎	صاد	शिकार करना	狩る	사냥하다	охотиться	cazar
113	hit	打	ضرب	टकराना	叩く	때리다	ударить	golpear
114	cut	切开	قطع	कटाई करना	切る	베다	резать	cortar
115	split	分开	شقّ	विभाजित करना	裂く	갈라지다	разделить	dividir
116	stab	扎	طعن	भोंकना	刺す	찌르다	заколоть	apuñalar
117	scratch	挠	خدش	खरोंचना	掻く	긁다	чесать	arañar
118	dig	挖	حفر	खोदना	彫る	파다	копать	cavar
119	swim	游泳	سبح	तैरना	泳ぐ	헤엄치다	плавать	nadar
120	fly (verb)	飞	طار	उड़ना	跳ぶ	날다	летать	volar
121	walk	走	مشى	टहलना	歩く	걷다	ходить	andar
122	come	来	أتى	आना	来る	오다	приходить	venir

123	lie (as in a bed)	躺	رقد	लेटना	嘘を吐く	눕다	лежать	acostarse
124	sit	坐	جلس	बैठना	座る	앉다	сидеть	sentarse
125	stand	站	وقف	खड़े होना	立つ	서다	стоять	ponerse de pie
126	turn	转	التفت	मुड़ना	回る	돌다	поворачивать	girar
127	drop	落(luo)下	سقط	गिराना, (बूंद)	落ちる（落とす）	떨어지다	ронять	caer
128	give	给	أعطى	देना	あげる	주다	давать	dar
129	hold	拿	أمسك	पकड़ना	取る	붙잡다	держать	apoyar
130	squeeze	挤压	عصر	निचोड़ना	搾る	짜다	сжимать	estrujar
131	rub	搓	مسح	रगड़ना	擦る	문지르다	тереть	frotar
132	wash	洗	غسل	धोना	洗う	씻다	мыть	lavar
133	wipe	擦	محا	पोंछना	拭く	닦다	вытирать	enjugar
134	pull	拉	جر	खींचना	引く	당기다	тянуть	tirar
135	push	推	دفع	धकेलना	押す	밀다	толкать	empujar
136	throw	扔	رمى	फेंकना	投げる	던지다	бросать	arrojar
137	tie (verb)	系（动词）	ألقى	बाँधना	結ぶ	매다	связывать	atar
138	sew	缝	خاط	सिलाई करना	縫う	꿰매다	шить	coser
139	count	数（动词）	عدّ	गिनना	数える	세다	Считать （называть числа в последовательном порядке)	contar
140	say	说	قال	कहना	言う	말하다	говорить	decir
141	sing	唱	غنّى	गाना	歌う	노래하다	петь	cantar
142	play	玩	لعب	खेलना, (नाटक)	遊ぶ（弾く）	놀다	играть	jugar
143	float	漂浮	طفا	बहाना	浮く	뜨다	плыть	flotar
144	flow	流	تدفق	बहना	流れる	흐르다	течь	correr
145	freeze	结冰	جمد	जमना	凍る	얼다	замерзать	congelar

146	swell	膨胀	ورم	सूजना	膨らむ（腫れる）	붓다	набухать	hinchar
147	sun	太阳	شمس	सूरज	太陽	해	солнце	sol
148	moon	月亮	قمر	चंद्रमा	月	달	луна	luna
149	star	星星	نجم	तारा	星	별	звезда	estrella
150	water	水	ماء	पानी	水	물	вода	agua
151	rain	雨	مطر	बारिश	雨	비	дождь	lluvia
152	river	河	نهر	नदी	川	강	река	río
153	lake	湖	بحيرة	झील	池	연못	озеро	lago
154	sea	海	بحر	समुद्र	海	바다	море	mar
155	salt	盐	ملح	नमक	塩	소금	соль	sal
156	stone	石头	حجر	पत्थर	岩	돌	камень	piedra
157	sand	沙子	رمل	बालू	砂	모래	песок	arena
158	dust	灰尘	غبار	धूल	埃	먼지	пыль	polvo
159	earth	土地	أرض	पृथ्वी	土	땅	земля	tierra
160	cloud	云	سحاب	बादल	雲	구름	облако	nube
161	fog	雾	ضباب	कोहरा	霧	안개	туман	niebla
162	sky	天空	سماء	आकाश	空	하늘	небо	cielo
163	wind	风	ريح	हवा	風	바람	ветер	viento
164	snow	雪	ثلج	प्राकृतिक बर्फ़	雪	눈 (날씨)	снег	nieve
165	ice	冰	جليد	बर्फ़	氷	얼음	лед	hielo
166	smoke	烟	دخان	धुआँ	煙	연기	дым	humo
167	fire	火	نار	आग	火	불	огонь	fuego
168	ash	灰烬	رماد	राख	灰	재	зола	ceniza
169	burn	烧	احترق	जलना	燃える（燃やす）	타다	гореть	arder
170	road	道路	طريق	सड़क	道	길	дорога	calle
171	mountain	山	جبل	पहाड़	山	산	гора	montaña
172	red	红	أحمر	लाल	赤い	빨강	красный	rojo
173	green	绿	أخضر	हरा	緑	초록	зеленый	verde
174	yellow	黄	أصفر	पीला	黄色	노랑	желтый	amarillo

175	white	白	أبيض	सफ़ेद	白い	하양	белый	blanco
176	black	黑	أسود	काला	黒い	검정	черный	negro
177	night	夜晚	ليل	रात	夜	밤	ночь	noche
178	day	白天	نهار	दिन	日	낮	день	día
179	year	年	سنة	साल	年	년	год	año
180	warm	暖和	دافئ	गर्म	暖かい	따뜻하다	теплый	caliente
181	cold	冷	بارد	ठंडा	冷たい	차다	холодный	frío
182	full	满	مليء	पूरा	満杯	가득차다	полный	lleno
183	new	新	جديد	नया	新しい	새-	новый	nuevo
184	old	陈旧的	قديم	पुराना	古い	옛-	старый	viejo
185	good	好	جيد	अच्छा	良い	좋다	хороший	bueno
186	bad	坏	سيء	ख़राब	悪い	나쁘다	плохой	mal
187	rot	腐烂	عفِن	सड़ांध	腐る	썩다	гнилой	podrido
188	dirty	脏的	وسخ	गंदा	汚い	더럽다	грязный	sucio
189	straight	直的	مستقيم	सीधा	真っ直ぐ	곧다	прямой	derecho
190	round	圆的	دائري	गोल	丸い	둥글다	круглый	redondo
191	sharp	锋利	حادّ	पैना	鋭い	날카롭다	острый	afilado
192	blunt	钝	غيْر حادّ	कुंद	鈍い	무디다	тупой	desafilado
193	smooth	光滑	ناعم	चिकना	滑らか（順調な、流暢な）	부드럽다	гладкий	suave
194	wet	湿的	رطب	गीला	濡れる	젖다	мокрый	mojado
195	dry	干的	جاف	सूखा	乾く	마르다	сухой	seco
196	correct	正确	صحيح	सही	正解（正す）	맞다	правильный	correcto
197	near	近	قريب	समीप	近い	가깝다	близкий	cerca
198	far	远	بعيد	दूर	遠い	멀다	далекий	distante
199	right (as opposed to left)	右	يَمِين	दायां , (सही)	右	오른쪽	правый	derecha
200	left	左	يَسار	बायां, (छोड़ा)	左	왼쪽	левый	izquierda

201	at	在	عند	पर	で	-에서	у (около)	a
202	in	在…里面	فِي	में	中	-안에	в	adentro
203	with	跟	مع	के साथ	と	-와	с	con
204	and	与	و	और	そして	그리고	и	y
205	if	如果	اذا	यदि	もし	만약--<br>한다면	если	si
206	because	因为	لأنّ	क्योंकि	だから	때문에	потому что	porque
207	name	名字	اِسْم	नाम	名前（タイトル）	이름	имя	nombre

Table S7. Profiles of two major climate PCs on 19 biologically meaningful climate variables

Climate variable	Variable description	PC1 loadings	PC2 loadings
BIO1	Annual Mean Temperature	-.97	-.06
BIO2	Mean Diurnal Range (Mean of monthly (max temp - min temp))	-.28	-.81
BIO3	Isothermality (BIO2/BIO7) (* 100)	-.87	.14
BIO4	Temperature Seasonality (standard deviation *100)	.69	-.57
BIO5	Max Temperature of Warmest Month	-.73	-.61
BIO6	Min Temperature of Coldest Month	-.89	.31
BIO7	Temperature Annual Range (BIO5-BIO6)	.53	-.74
BIO8	Mean Temperature of Wettest Quarter	-.49	-.45
BIO9	Mean Temperature of Driest Quarter	-.84	.09
BIO10	Mean Temperature of Warmest Quarter	.82	.49
BIO11	Mean Temperature of Coldest Quarter	.94	.20
BIO12	Annual Precipitation	-.48	.64
BIO13	Precipitation of Wettest Month	-.67	.24
BIO14	Precipitation of Driest Month	.33	.85
BIO15	Precipitation Seasonality (Coefficient of Variation)	-.62	-.59
BIO16	Precipitation of Wettest Quarter	-.66	.30
BIO17	Precipitation of Driest Quarter	.26	.89
BIO18	Precipitation of Warmest Quarter	-.14	.34
BIO19	Precipitation of Coldest Quarter	-.09	.81

Supplementary References

1 Cole, M. W., Bassett, D. S., Power, J. D., Braver, T. S. & Petersen, S. E. Intrinsic and task-evoked network architectures of the human brain. *Neuron* **83**, 238–251 (2014).

2 Romney, A. K., Moore, C. C., Batchelder, W. H. & Hsia, T.-L. Statistical methods for characterizing similarities and differences between semantic structures. *Proceedings of the National Academy of Sciences* **97**, 518–523 (2000).

3 Van Paridon, J. & Thompson, B. subs2vec: Word embeddings from subtitles in 55 languages. *Behavior Research Methods* **53**, 629–655 (2021).

4 Chen, G., Taylor, P. A., Shin, Y.-W., Reynolds, R. C. & Cox, R. W. Untangling the relatedness among correlations, Part II: Inter-subject correlation group analysis through linear mixed-effects modeling. *NeuroImage* **147**, 825–840 (2017).

5 Malik-Moraleda, S. *et al.* An investigation across 45 languages and 12 language families reveals a universal language network. *Nature Neuroscience* **25**, 1014–1019 (2022).

6 Binder, J. R. *et al.* Toward a brain-based componential semantic representation. *Cognitive Neuropsychology* **33**, 130–174 (2016).
