# Supplementary material for: Semantic similarity across languages reflects neurocognitive dimensions shaped by climate
Source: Nat Commun. 2026 Mar 16;17:4016. doi: 10.1038/s41467-026-70608-8 (PMC13136401; doi:10.1038/s41467-026-70608-8)
Supplement: Supplementary file 2 — Reporting Summary [file 41467_2026_70608_MOESM2_ESM.pdf]

nature portfolio

Corresponding author(s): Yanchao Bi; Xiaosha Wang

Last updated by author(s): Jan 28, 2026

Reporting Summary

Nature Portfolio wishes to improve the reproducibility of the work that we publish. This form provides structure for consistency and transparency in reporting. For further information on Nature Portfolio policies, see our Editorial Policies and the Editorial Policy Checklist.

Statistics

For all statistical analyses, confirm that the following items are present in the figure legend, table legend, main text, or Methods section.

n/a	Confirmed	
☐	☒	The exact sample size (n) for each experimental group/condition, given as a discrete number and unit of measurement
☐	☒	A statement on whether measurements were taken from distinct samples or whether the same sample was measured repeatedly
☐	☒	The statistical test(s) used AND whether they are one- or two-sided<br>*Only common tests should be described solely by name; describe more complex techniques in the Methods section.*
☐	☒	A description of all covariates tested
☐	☒	A description of any assumptions or corrections, such as tests of normality and adjustment for multiple comparisons
☐	☒	A full description of the statistical parameters including central tendency (e.g. means) or other basic estimates (e.g. regression coefficient) AND variation (e.g. standard deviation) or associated estimates of uncertainty (e.g. confidence intervals)
☐	☒	For null hypothesis testing, the test statistic (e.g. F, t, r) with confidence intervals, effect sizes, degrees of freedom and P value noted<br>*Give P values as exact values whenever suitable.*
☒	☐	For Bayesian analysis, information on the choice of priors and Markov chain Monte Carlo settings
☒	☐	For hierarchical and complex designs, identification of the appropriate level for tests and full reporting of outcomes
☐	☒	Estimates of effect sizes (e.g. Cohen's d, Pearson's r), indicating how they were calculated

Our web collection on statistics for biologists contains articles on many of the points above.

Software and code

Policy information about availability of computer code

Data collection	Study 1: Multilingual word embeddings were obtained from publicly available pre-trained models (fastText, subs2vec). Lexical data were extracted from NorthEuraLex and CLICS databases. Environmental variables were obtained from WorldClim and D-PLACE databases.<br><br>Study 2: Behavioral semantic ratings were collected online from native speakers (N = 253 participants across 8 languages) recruited via Appen (https://www.appen.com). Participants rated 207 concepts on 13 semantic dimensions using Likert scales.<br><br>Study 3: fMRI data were obtained from the publicly shared dataset by Malik-Moraleda et al. (2022). Language-level neural representational dissimilarity matrices were computed from these data using correlational distance.
Data analysis	All data analyses were conducted using Python (version 3.9.1) and R (version 4.5.2). Complete details of software packages and analysis code are available at OSF (https://doi.org/10.17605/OSF.IO/SUYEB).

For manuscripts utilizing custom algorithms or software that are central to the research but not yet described in published literature, software must be made available to editors and reviewers. We strongly encourage code deposition in a community repository (e.g. GitHub). See the Nature Portfolio guidelines for submitting code & software for further information.

Data

Policy information about availability of data

All manuscripts must include a data availability statement. This statement should provide the following information, where applicable:

- Accession codes, unique identifiers, or web links for publicly available datasets
- A description of any restrictions on data availability
- For clinical datasets or third party data, please ensure that the statement adheres to our policy

The multilingual fastText embeddings used in Study 1 are publicly available at https://fasttext.cc. Additional distributional semantic vectors used in validation analyses were obtained from the subs2vec model (https://github.com/jvparidon/subs2vec). The NorthEuraLex word list and concept translations are available at http://northeuralex.org. Colexification data were obtained from the CLICS database (version 3.0; https://clics.clld.org). Climate variables were obtained from the WorldClim 2.0 database (https://www.worldclim.org). Cultural variables were obtained from the D PLACE database (https://d-place.org). The behavioral rating data from Study 2 (13 dimensional ratings for 207 concepts in 8 languages), together with derived semantic matrices and environmental distance matrices, are available at OSF (https://osf.io/suyeb). The fMRI data re analyzed in Study 3 were originally collected and shared by Malik Moraleda et al. (2022) and are available at OSF (https://osf.io/cw89s). Our derived language level neural representational dissimilarity matrices and analysis scripts are available at OSF (https://osf.io/suyeb). Source Data underlying the main figures and tables are provided with this paper.

Research involving human participants, their data, or biological material

Policy information about studies with human participants or human data. See also policy information about sex, gender (identity/presentation), and sexual orientation and race, ethnicity and racism.

Reporting on sex and gender	Study 1 used the language level data; For Study 2, the 253 participants were analyzed in the main texts (gender: male = 113, female = 139, other = 1). Sex information was not collected. Gender was used as one of the demographic covariates in the analyses; Study 3 used dataset collected and shared by Malik Moraleda et al. (2022).
Reporting on race, ethnicity, or other socially relevant groupings	Study 1 used the language level data; For Study 2, participants were recruited from 58 city sites across 8 different countries/ languages: Arabic (Egypt), Chinese (China), English (USA), Hindi (India), Japanese (Japan), Korean (South Korea), Russian (Russia), and Spanish (Spain). Other socially relevant groupings were collected and deposited to OSF; Study 3 used dataset collected and shared by Malik Moraleda et al. (2022)
Population characteristics	For Study 2, native language speakers with diverse geographic and linguistic backgrounds. Mage = 35.76, SDage = 11.87. The demographic information were used as covariates in the analyses.
Recruitment	For Study2, we recruited participants from the participant pools on the Appen Butler Hill crowdsourcing platform. No additional demographic characteristics were required during the recruitment processes.
Ethics oversight	This study was approved by the Institutional Review Board State of the Key Laboratory of Cognitive Neuroscience and Learning, Beijing Normal University.

Note that full information on the approval of the study protocol must also be provided in the manuscript.

Field-specific reporting

Please select the one below that is the best fit for your research. If you are not sure, read the appropriate sections before making your selection.

☐ Life sciences ☒ Behavioural & social sciences ☐ Ecological, evolutionary & environmental sciences

For a reference copy of the document with all sections, see nature.com/documents/nr-reporting-summary-flat.pdf

Behavioural & social sciences study design

All studies must disclose on these points even when the disclosure is negative.

Study description	Our data are quantitative with mixed-methods components (computational modeling, behavioral experiments, and neuroimaging analysis). Study 1 was performed at the language level, deriving such semantic representations based on large text computations that allow for analyses over billions of language texts across 53 languages (10 language families), where we recovered the words' semantic effective dimensions in each model with the computed semantic projection approach using embedding data in each language. Study 2 was performed at the individual subject level, deriving words'neurocognitive semantic representation from individual subjective ratings collected from participants from diverse language and cultural settings (N = 253; from 58 city sites across 8 selected countries with varying climate and cultural properties). Study 3 was performed at the individual neural activity level, using neural data from participants from diverse language and cultural settings (N=86; 45 languages).
Research sample	Study 1 sampled 53 languages (10 language families). Two primary criteria were used in the sample selection process. First, the chosen languages were sourced from the NorthEuralex (NEL) dataset and had translated word forms accessible in the fast-text word embedding models. Study 2 sampled 272 online participants from 58 city sites across 8 different countries/languages. Nineteen participants were excluded from further analyses because the correlation between their rating vector and the averaged group vector within each language was lower than 0.5. The final sample size was 253 participants and ranged from 30 to 34 participants for each

	country (self-reported gender: 113 male, 139 female, 1 other/preferred not to say; age range: 18-83 years). The dataset in Study 3 comprises neural activity measurements during a language comprehension task performed by 86 native speakers across 45 languages from 12 language families. Eighty-six participants (43 males; age range: 19-45 years) were recruited from Boston, the United States.
Sampling strategy	The convenient samples were adopted. No prior sample-size calculation was performed. The sample sizes were based on those used for previous cross-culture comparisons (5-20 languages; 10-30 participants per language) .
Data collection	For language text study, we use the pretrained word embedding models accessible through fastText archives. For human participant study, we collected the data from the Appen Butler Hill crowdsourcing platform (http://www.appen.com).
Timing	The human participant data were collected between April 2023 and July 2023.
Data exclusions	See "Research sample".
Non-participation	No participations dropped out.
Randomization	Investigators were not blinded to group assignments, as language identity was a key variable of interest across all studies. No randomization for Study 1 and Study 3. For human participant study (Study 2), the order of words across the three sessions was randomly assigned to each participant from 30 wordlists created by pseudorandom shuffling of the 207 concepts.

Reporting for specific materials, systems and methods

We require information from authors about some types of materials, experimental systems and methods used in many studies. Here, indicate whether each material, system or method listed is relevant to your study. If you are not sure if a list item applies to your research, read the appropriate section before selecting a response.

Materials & experimental systems

n/a	Involved in the study
☒	☐ Antibodies
☒	☐ Eukaryotic cell lines
☒	☐ Palaeontology and archaeology
☒	☐ Animals and other organisms
☒	☐ Clinical data
☒	☐ Dual use research of concern
☒	☐ Plants

Methods

n/a	Involved in the study
☒	☐ ChIP-seq
☒	☐ Flow cytometry
☒	☐ MRI-based neuroimaging

Plants

Seed stocks	*Report on the source of all seed stocks or other plant material used. If applicable, state the seed stock centre and catalogue number. If plant specimens were collected from the field, describe the collection location, date and sampling procedures.*
Novel plant genotypes	*Describe the methods by which all novel plant genotypes were produced. This includes those generated by transgenic approaches, gene editing, chemical/radiation-based mutagenesis and hybridization. For transgenic lines, describe the transformation method, the number of independent lines analyzed and the generation upon which experiments were performed. For gene-edited lines, describe the editor used, the endogenous sequence targeted for editing, the targeting guide RNA sequence (if applicable) and how the editor was applied.*
Authentication	*Describe any authentication procedures for each seed stock used or novel genotype generated. Describe any experiments used to assess the effect of a mutation and, where applicable, how potential secondary effects (e.g. second site T-DNA insertions, mosiacism, off-target gene editing) were examined.*
